# Supplementary material for: Different Role of Bisulfite/Sulfite in UVC-S(IV)-O2 System for Arsenite Oxidation in Water
Source: Molecules. 2019 Jun 21;24(12):2307. doi: 10.3390/molecules24122307 (PMC6631152; doi:10.3390/molecules24122307)
Supplement: Supplementary file 1 [file molecules-24-02307-s001.pdf]

Supporting information

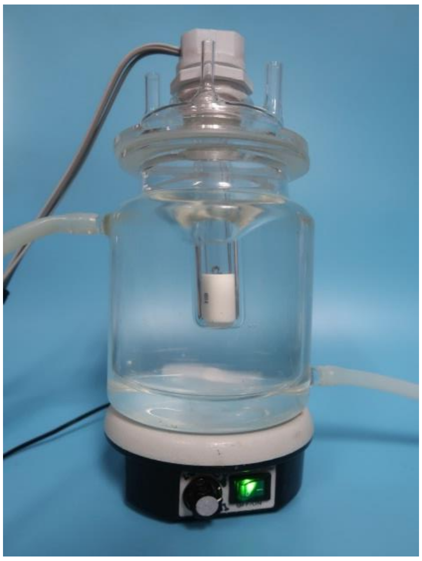

Fig.S1 the experimental setup used by UVC-S(IV)-O_2 system.

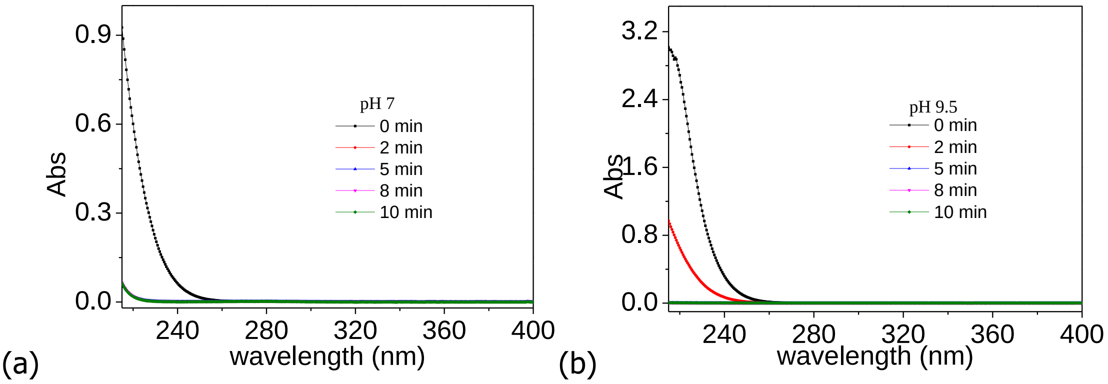

Fig. S2. The changes of S(IV) spectrum in the UVC-S(IV)-O_2 system during reaction time. Initial conditions: $[S(IV)]_0$ = 2 mM, $[As(III)]_0$ = 5 μM. pH 7 or 9.5.

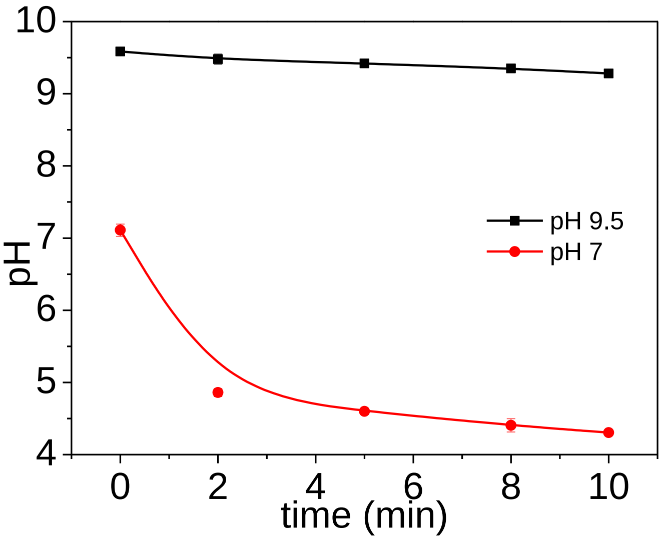

Fig. S3. The changes of pH in the UVC-S(IV)-O_2 system during reaction time. Initial conditions: $[S(IV)]_0$ = 2 mM, $[As(III)]_0$ = 5 μM. pH 7 or 9.5.

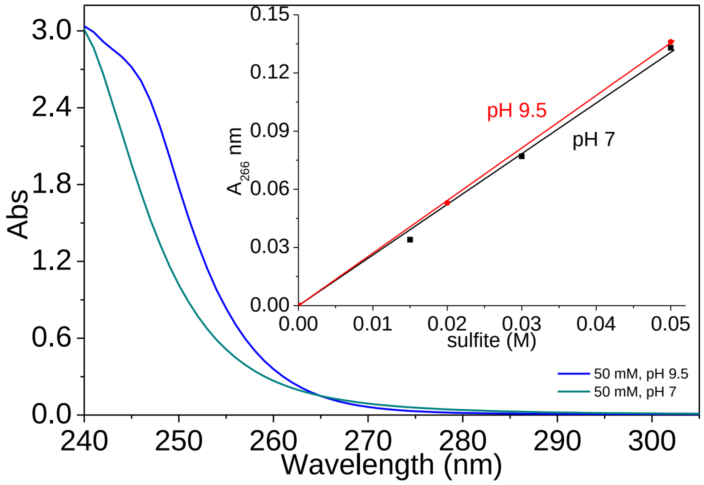

Fig. S4. Absorption spectra of 50 mM sulfite at pH 7 and 9.5 in 1 cm optical cell. Insert – the dependence of absorbance at 266 nm upon S(IV) for pH 7 and pH 9.5.

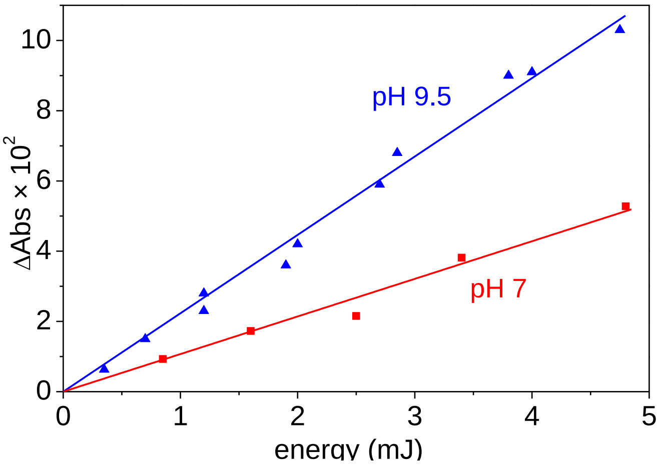

Fig. S5. Dependence of the yield of e_{aq}^- absorbance at 720 nm upon energy of excitation at pH 7 and 9.5.
